# Direct Effect of Chenodeoxycholic Acid on Differentiation of Mouse Embryonic Stem Cells Cultured under Feeder-Free Culture Conditions

**DOI:** 10.1155/2013/375076

**Published:** 2012-12-29

**Authors:** Soon-Jung Park, Seul-Bi Lee, Dong-Sup Lee, Young-Joon Ryu, Gene Lee, Jaejin Cho

**Affiliations:** ^1^Lab of Developmental Biology and Stem Cell Differentiation/Transplantation, School of Dentistry, Seoul National University, 28 Yongun-dong, Chongno-gu, Seoul 110-749, Republic of Korea; ^2^Dental Research Institute, School of Dentistry, Seoul National University, 28 Yongun-dong, Chongno-gu, Seoul 110-749, Republic of Korea; ^3^Department of Biomedical Sciences, College of Medicine, Seoul National University, 28 Yongun-dong, Chongno-gu, Seoul 110-799, Republic of Korea; ^4^Department of Pathology, College of Medicine, University of Ulsan and Asan Medical Center, 88 Olympic-ro 43-gil, Songpa-gu, Seoul 138-736, Republic of Korea

## Abstract

Chenodeoxycholic acid (CDCA), a farnesoid X receptor (FXR) ligand, is a member of the nuclear receptor family and is probably involved in regulating the cellular activities of embryonic stem (ES) cells. Recently, although it was reported that the FXR ligand can mediate differentiation, apoptosis, and/or growth arrest in several cell types, it is still not well known how CDCA mediates effects in ES cells. Therefore, we investigated the direct effect of CDCA on mES cells. Feeder-free mES cells were treated in a dose-dependent manner with CDCA (50, 100, and 200 **μ**M) for 72 h, and then a 100 **μ**M CDCA treatment was performed for an additional 72 h. We analyzed the morphology, cell growth, cell characteristics, immunocytochemistry, and RT-PCR. In CDCA-treated cells, we observed the disappearance of pluripotent stem cell markers including alkaline phosphatase, Oct4, and Nanog and a time- and dose-dependent increase in expression of nestin, PAX6, and **α**-smooth muscle actin, but not **α**-fetoprotein. The 100 **μ**M CDCA-treated cells in their second passage continued this differentiation pattern similar to those in the controls. In conclusion, these results suggest that CDCA can guide mES cells by an FXR-independent pathway to differentiate into ectoderm and/or mesoderm, but not endoderm.

## 1. Introduction

Since the establishment of embryonic stem (ES) cell lines [1, 2], it has been known that ES cells have the capacity for self-renewal and pluripotency, with the ability to differentiate into multiple cell types *in vitro *and *in vivo.* These characteristics of ES cells make them a valuable model system for differentiation study and cell-based regeneration therapies.

Numerous reports have documented the differentiation of ES cells into specific cell types, such as neurons [3], cardiomyocytes [4], adipocytes [5], endothelial cells [6], hepatocytes [7], keratinocytes [8], and pancreatic cells [9] under the appropriate culture conditions. So far, ES cell differentiation required the formation of an embryoid body (EB) in most studies in general. However, alternative approaches have shown directed differentiation of ES cells into a desired lineage without going through EB formation [10, 11]. There are some problems in ES cell differentiation through EB formation. It may lead to uncontrollable complexity and to unwanted cell types [12], and some of the cells of the EB might not be terminally differentiated [10].

The farnesoid X receptor (FXR, NR1H4), meanwhile, may modulate the differentiation into myocyte [13] during myogenesis of tissue-specific stem cells. Therefore, the differentiated cell population tends to be directed more uniform, and a larger number of precursors and more differentiated cells can be obtained using this pathway. The FXR, a member of the nuclear receptor superfamily, is highly expressed in liver, intestine, and kidney tissues [14]. FXR is known to be a key player in the control of multiple metabolic pathways including bile acid biosynthesis from cholesterol and lipid/glucose metabolism [15, 16]. In liver, especially, activated FXR induces liver regeneration by a homeostatic mechanism [17] and affects vascular remodeling [18]. In the intestine, it protects the tissue from bacterial-induced mucosal injury by bile acids [19]. It is also known that the FXR activators inhibit cell proliferation, trigger differentiation, and induce apoptosis. Bile acids reduce the growth of keratinocytes, human fibroblasts, and smooth muscle cells [20–22]. Additionally, activated FXR plays a critical role in regulating adipogenesis [23] and also induces apoptosis in cancer cells [24]. However, studies on the effects of activated FXR on proliferation or differentiation of ES cells are scarce.

Chenodeoxycholic acid (CDCA, 3*α*, 7*α*-dihydroxy-5*β*-cholanic acid) is a primary bile acid directly synthesized from cholesterol. It was shown to be the most potent activator of the FXR [25, 26]. It binds directly to FXR, which then regulates several known FXR target genes and induces bile acid-binding protein for bile acid transport [27]. Therefore, CDCA is not simply metabolic products but also regulates involving gene transcription and signaling of transduction pathway. Moreover, CDCA is involved in many cellular activities including cell proliferation, differentiation, and apoptosis [23, 28].

In this paper, we investigated the effects of the FXR ligand and CDCA on the differentiation of mouse ES (mES) cells without a feeder layer or EB formation. To examine whether CDCA mediates differentiation through FXR signaling,we checked the mRNA expression of FXR in CDCA-induced and differentiated mES cells. Additionally, direct differentiation was performed in the presence of LIF in order to determine the relationship between LIF signaling and CDCA-mediated cellular activities. 

## 2. Materials and Methods

### 2.1. Mouse ES Cell Cultures

The E14TG2a (ATCC number CRL-1821) mouse ES (mES) cell line was routinely cultured on feeder layers of primary mouse embryonic fibroblasts (MEF) pretreated with mitomycin C. The cells were maintained with Dulbecco's modified Eagle's medium (DMEM; Welgene, Daegu, Republic of Korea) supplemented with 15% fetal bovine serum (FBS; HyClone Laboratories, Logan, UT, USA), 0.1 mM 2-mercaptoethanol (Sigma-Aldrich Corp, St. Louis, MO, USA), 1% nonessential amino acids (NEAA; Gibco-Invitrogen, Carlsbad, CA, USA), 1% (v/v) penicillin-streptomycin (Gibco-Invitrogen), and 1,000 U/mL of recombinant mouse leukemia inhibitory factor (LIF; Chemicon, Temecula, CA, USA) in 5%  CO_2_ at 37°C. The mES cells were subcultured onto new feeders every 3 or 4 days using 0.05% trypsin-EDTA (Gibco-Invitrogen).

### 2.2. Treatment of mES Cells with Chenodeoxycholic Acid (CDCA)

For direct differentiation of mES cells by CDCA, feeder cells were removed by plating on nongelatin coated dish for 1 h, which allowed the feeder cells to adhere, while most of the mES cells stayed in the suspension. The suspended mES cells were once transferred onto a new 0.1% gelatin-coated dish for propagation in the presence of 1,000 U/mL of LIF. The feeder-free mES cells were subcultured after 24 h. Then the cells were incubated under different conditions for 72 h. The cells were incubated in (i) basal medium (spontaneously differentiated control), (ii) basal medium supplemented with 1,000 U/mL of LIF (undifferentiated control), and (iii) basal medium supplemented with 0.1% dimethyl sulfoxide (DMSO; Sigma-Aldrich Corp) in the absence of LIF (solvent control). Treated groups were cultured in basal medium supplemented with (iv) 50 *μ*M, (v) 100 *μ*M, and (vi) 200 *μ*M of CDCA. After the treatment for 72 h, the control groups and 100 *μ*M CDCA-treated cells were subcultured using 0.05% trypsin-EDTA (Gibco-Invitrogen) on new gelatinized dishes and treated with 100 *μ*M CDCA for additional 72 h.

The cells were incubated in basal medium, basal medium supplemented with 1,000 U/mL of LIF, and basal medium supplemented with 0.1% dimethyl sulfoxide (DMSO; Sigma-Aldrich) as a control. Treated groups were cultured in basal medium supplemented with 50, 100, and 200 *μ*M of chenodeoxycholic acid (CDCA, Sigma-Aldrich) for 72 h. Then, the 100 *μ*M CDCA-treated cells were subcultured and treated with 100 *μ*M CDCA for an additional 72 h. The cell number was counted by trypan blue dye exclusion. The experiment was repeated three times. 

### 2.3. Cell Viability Assay

Cell viability was assessed by a tetrazolium salt (WST-1)-based colorimetric assay. A commercial WST-1 kit (EZ CyToX; Daeil Lab, Seoul, Republic of Korea) was used. The absorbance was measured at 450 nm Bio-Rad microplate reader Model-550 (Bio-Rad, Hercules, CA, USA).

### 2.4. Alkaline Phosphatase Activity

The cells were fixed with a 4% paraformaldehyde solution and stained with Naphthol/Fast Red Violet Solution (Mix Fast Red Violet (FRV) with Naphthol AS-BI phosphate solution and water in a 2 : 1 : 1 ratio) at room temperature in the dark for 15 min. Later, the cells were rinsed with PBS and observed under a phase contrast microscope. 

### 2.5. Immunocytochemistry

To detect pluripotent stem cell markers and three-germ layer-specific marker antigens, the cells were fixed in culture dishes with 4% paraformaldehyde in phosphate-buffered saline (PBS) (0.01 M, pH 7.4) for 15 min. Endogenous peroxidase activity was blocked by hydrogen peroxide for 30 min at RT after a PBS wash. The cells were permeabilized with 0.1% Triton X-100 for 10 min and incubated with normal goat serum (Jackson Immunoresearch Laboratory, West Grove, PA) for 60 min to block nonspecific binding sites. The cells were incubated with the primary antibodies overnight at 4°C. The primary antibodies were Oct4 (Santa Cruz Biotechnology, Santa Cruz, CA, USA) and Nanog (Abcam, UK) as pluripotent stem cell markers, nestin (Chemicon) and PAX6 (Chemicon) as ectoderm markers, *α*-smooth muscle actin (Sigma-Aldrich) as mesoderm marker, and *α*-fetoprotein (Santa Cruz) as endoderm marker. The cells were then probed with secondary antibodies (peroxidase-labeled goat anti-mouse IgG or goat anti-rabbit IgG (1 : 200); Jackson Immunoresearch Laboratory) for 1 h at RT. DAB (DAKO, Carpinteria, CA, USA) was used for visualization about 30 seconds.

### 2.6. Total RNA Isolation and RT-PCR

Total RNA was extracted from the cells using TRIzol (Gibco-Invitrogen) according to the manufacturer's instructions. After total RNA extraction, the cells were treated with DNAse I (Rnase free, Takara, Japan) for discarding genomic DNA contamination. The concentration and quality of isolated RNA were determined using an ND-1000 Spectrophotometer (NanoDrop Technologies, USA). Complementary DNA was synthesized from 1 *μ*g of total RNA using M-MLV Reverse Transcriptase Kit I (Bioneer, Daejon, Republic of Korea) with oligo-dT primers. One microliter of cDNA was used as template in the PCR reactions. Each PCR reaction mixture contained PCR buffer (2.0 mM MgCl_2_), 2.5 mM of each dNTP, 10 pM of each mouse-specific primer sets, one unit of i-MAX II DNA Polymerase (Intron, Seoul, Republic of Korea).The primer sets and the PCR conditions are summarized in Table 1. The PCR products were analyzed by electrophoresis on a 1.5% agarose gel containing 0.4 *μ*g/mL ethidium bromide (Sigma-Aldrich Corp). Band intensities were quantified three times each by densitometry analysis using Bio1D software (Vilber Lourmat, Mame la Vallee, France).

## 3. Results

### 3.1. Morphological Changes and Viability of CDCA-Treated mES Cells

The attached feeder-free mES cells formed tightly packed colonies, same as cells on feeder layers. The nucleus shows irregularly shaped thin smooth nuclear membrane with prominent multiple nucleoli. The cytoplasm is scanty in volume and cytoplasmic molding in shape. Feeder-free mES cells still showed the characteristics of pluripotent stem cells, in terms of their alkaline phosphatase activity, Oct4, and Nanog expression (see Supplement 1 in Supplementary Material available online at doi:10.1155/2012/375076). This result indicates that mES cells can be maintained in an undifferentiated state in feeder-free culture conditions.

**Figure 1:**
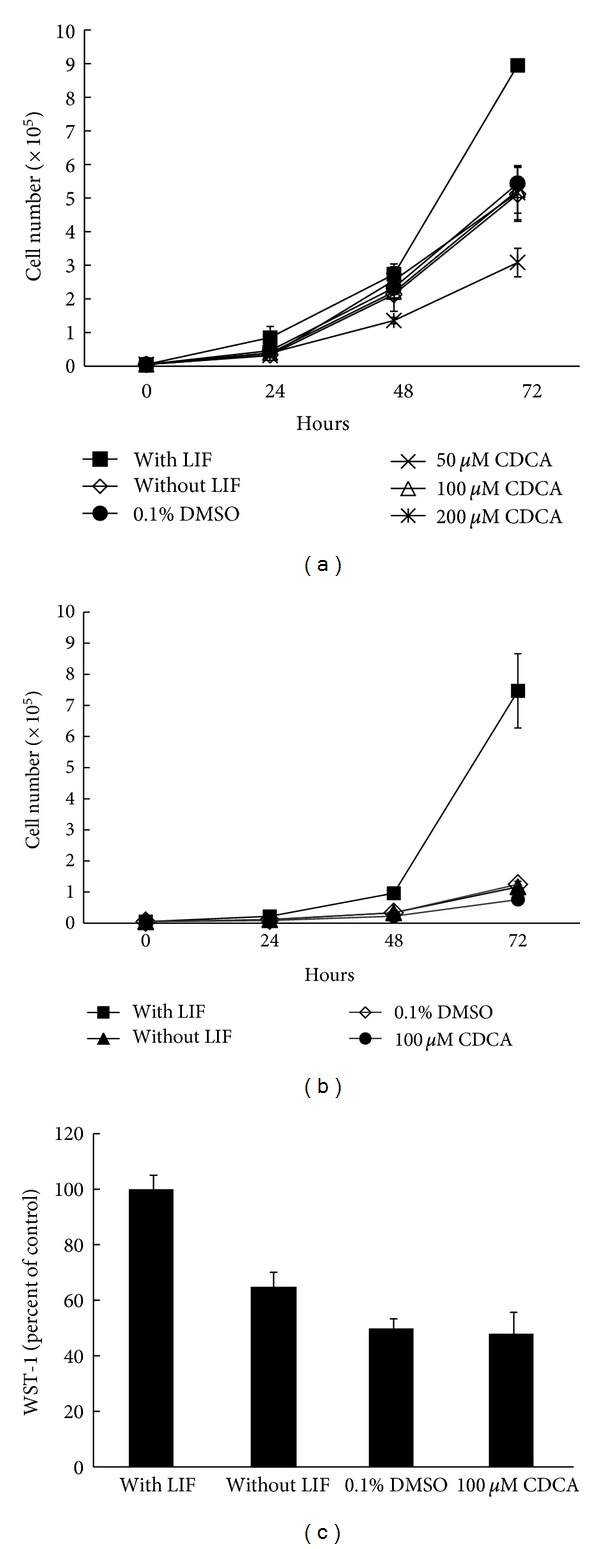
Growth rate and cell viability of CDCA-treated mES cells. Changes in mES cell number after CDCA treatment (a) and after the second round of treatment with 100 *μ*M CDCA (b). The effects of 100 *μ*M CDCA on the viability of subcultured mES cells after 72 h incubation, measured by the WST-1 assay. Data represent viability as a percentage of the control (1000 U/mL of LIF-treated cells) (c). Data are expressed as the mean ± SE (*n* = 3). LIF: leukemia inhibitory factor; DMSO: dimethyl sulfoxide; CDCA: chenodeoxycholic acid.

When LIF was discarded, the mES cells differentiated spontaneously. Moreover, the CDCA-treated cells showed remarkable changes. The differentiated cells showed decrease in nucleus size, abundant cytoplasm, and low nucleus/cytoplasm ratio, in a dose- and time-dependent manner. The prominent nucleoli are also decreased in size, and cytoplasmic pod extension was noted. We observed primarily this type of cells in the 50 *μ*M CDCA-treated group and more stretched sharp-ended cytoplasmic cells in the 100 *μ*M CDCA-treated group (Supplement 2). CDCA-treated cells became larger, flatter, and more elongated in a time-dependent manner. The high-dose CDCA-treated (200 *μ*M) group shows abundant and thin cytoplasm with vacuolation, similar to the senescence phenomenon. Furthermore, the treated cells had inhibited cell proliferation and the cell number was decreased by 43.25% compared to the vehicle control (Figure 1(a)). 

After subculturing, the mES cells treated with 100 *μ*M CDCA changed their morphology according to the previously described pattern. Treatment with 100 *μ*M CDCA reduced the cell number to the cell number seen in the negative controls (without LIF and 0.1% DMSO-treated group) compared to the positive control with LIF (Figure 1(b)). The cell viability of 100 *μ*M CDCA-treated cells, as measured by MTT assay, was similar to that of the negative controls. These results indicate that 100 *μ*M CDCA does not have a cytotoxic effect on mES cells grown under feeder-free conditions, even after a 2nd passage. In addition, adding LIF to the medium clearly stimulates the mES cells under feeder-free conditions (Figure 1(c)).

### 3.2. Characterization of CDCA-Treated mES Cells

**Figure 2:**
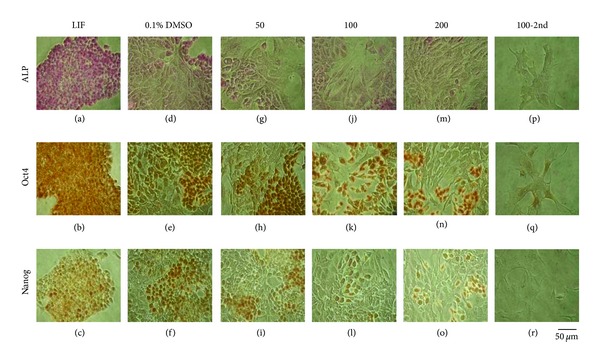
Changes of pluripoent markers in CDCA-treated mES cells. mES cells were cultivated with 50 *μ*M, 100 *μ*M, or 200 *μ*M CDCA for 72 h. Subcultured mES cells were treated with 100 *μ*M CDCA for another 72 h. The cells were probed with antibodies against pluripotent stem cell markers, alkaline phosphatase (ALP), Oct4, and Nanog. LIF: leukemia inhibitory factor; DMSO: dimethyl sulfoxide; CDCA: chenodeoxycholic acid

**Figure 3:**
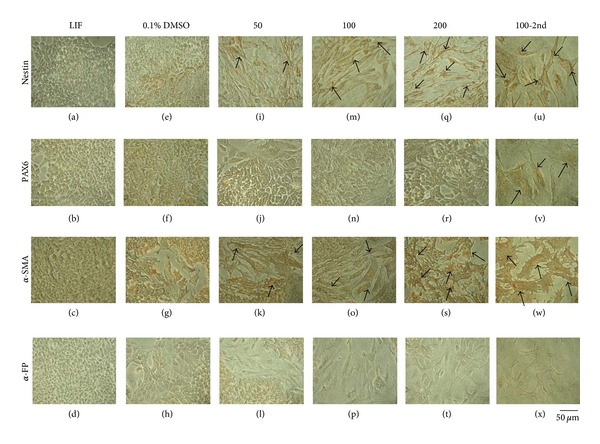
Characteristic changes in CDCA-treated mES cells. mES cells were cultivated with 50 *μ*M, 100 *μ*M, or 200 *μ*M CDCA for 72 h. Subcultured mES cells were treated with 100 *μ*M CDCA for another 72 h. Lineage-specific markers such as nestin and PAX6, *α*-smooth muscle actin (*α*-SMA), and *α*-fetoprotein (*α*-FP). LIF: leukemia inhibitory factor; DMSO: dimethyl sulfoxide; CDCA: chenodeoxycholic acid.

The expression of ALP, Oct4, and Nanog was lower in LIF-withdrawn mES cells and DMSO-treated cells than in LIF-treated mES cells. CDCA treatment reduced the expression of ALP, Oct4, and Nanog in a concentration-dependent manner, even when compared to negative controls. ALP, Oct4, and Nanog expression in LIF control showed almost 100% positive cells. CDCA decreased the ALP expression about 3–5% positive cells. These phenomena were similar to Nanog expression. In contrast, decrease of Oct4 expression by CDCA was much slower than ALP and Nanog. 50 *μ*M CDCA reduced Oct4-positive cells by 70% compared to LIF control. The decreasing effect showed CDCA dose-dependent manner (100 *μ*M: 50%, 200 *μ*M: 20%). Oct4 positive cells almost disappeared but still remained at the second passage of mES cells treated by 100 *μ*M CDCA, respectively, (Figure 2). While the cells in the control groups were negative for all three germ layer markers, CDCA-treated mES cells showed strong expression of nestin and *α*-smooth muscle actin. CDCA increased nestin expression in concentration-dependent manner (50 *μ*M: 20%, 100 *μ*M: 50%, 200 *μ*M: 70%). The second passage of 100 *μ*M CDCA showed almost 90% cells showing positive. CDCA increased Pax6 expression, but the pattern was much slower than nestin. At the second passage of 100 *μ*M CDCA-treated mES cells showed positive cells by 50%, respectively. Subcultured mES cells were treated with 100 *μ*M CDCA for another 72 h. CDCA induced the *α*-smooth muscle actin expression in lower concentration compared to nestin and Pax6. From 50 *μ*M CDCA treatment, it showed strong expression. However, *α*-fetoprotein was not detected in any CDCA-treated groups (Figure 3). 

**Figure 4:**
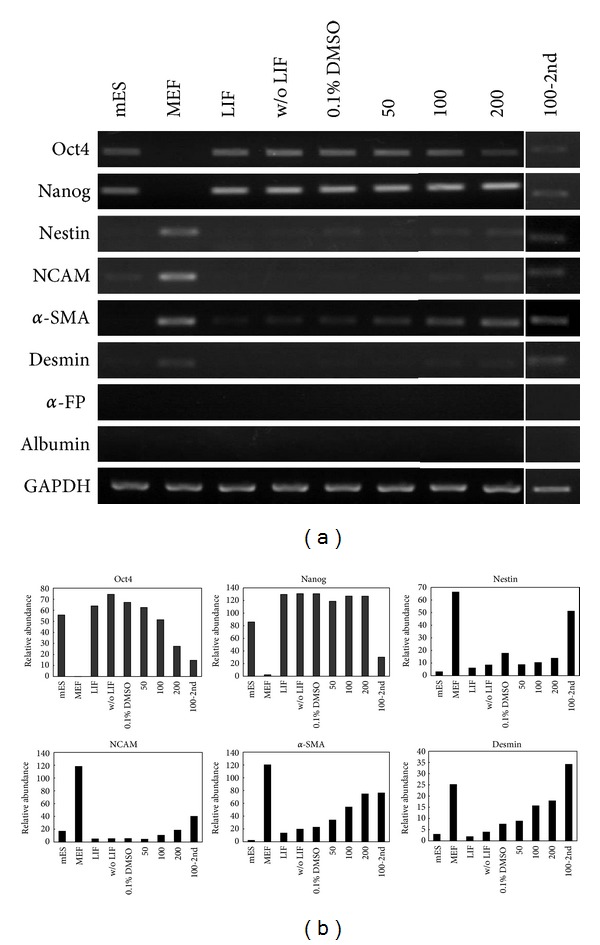
Characterization of CDCA-treated mES cells by RT-PCR analysis. (a) The mRNA expression of pluripotent stem cell markers and lineage specific makers were analyzed. The mES cells were cultivated with 50 *μ*M, 100 *μ*M, or 200 *μ*M CDCA for 72 h. Sub-cultured mES cells were treated with 100 *μ*M CDCA for another 72 h. MEF, mouse embryonic fibroblast, LIF, leukemia inhibitory factor; DMSO, dimethyl sulfoxide; CDCA, chenodeoxycholic acid. (b) Relative semi-quantitation of PCR signals by image analysis.

The mRNA expression pattern was similar to the protein expression pattern. In the 200 *μ*M CDCA-treated mES cells cultured for 72 h, Oct4 mRNA expression was downregulated and the phenomenon disappeared at 100 *μ*M CDCA for the second passage (total 144 h incubation with CDCA). Nanog mRNA expression was maintained at a steady state, but it disappeared similar to Oct4 at 100 *μ*M CDCA for the second passage. Cells treated with CDCA for 72 h expressed nestin, NCAM, *α*-smooth muscle actin, and desmin at 200 *μ*M CDCA. The pattern was clearer at 100 *μ*M CDCA for the second passage. However, *α*-fetoprotein and albumin were not expressed, which is consistent with the results of the immunocytochemical analysis (Figures 4(a) and 4(b)). FXR mRNA was not detected in any CDCA-treated cells or control group cells (Supplement 3).

## 4. Discussion

In this study, we investigated the effect of CDCA, an FXR ligand, on mES cell differentiation under feeder-free conditions. We focused on the effect of CDCA on the proliferation and differentiation of mES cells into specific lineages by analyzing the changes in mES cell characteristics, mRNA expression patterns, and cell physiology. First, we treated mES cells cultured without a feeder layer with different doses of CDCA in the culture medium for 72 h. Because 100 *μ*M CDCA showed an effect on the differentiation of mES cells without changing cell viability, when compared to the controls, we treated the cells for an additional 72 h. The second round treatment of mES cells with CDCA resulted in ectodermal and mesodermal differentiation, but not endodermal lineage. During differentiation, the cell viability was similar to that observed for the negative controls (0.1% DMSO without LIF). 

In our preliminary experiments, we changed the mES cell culture to feeder-free culture by adding 1,000 IU LIF. The cells were successfully maintained, but the margin of the colony was occasionally irregular. All of the ES cell characteristics were maintained, as previously reported elsewhere [29]. 

Since the regulation of the nuclear receptor, and especially FXR-dependent bile acid signaling, could contribute to endodermal organ and liver regeneration [17], there is a possibility that the FXR ligand, CDCA, could cause differentiation of mES cells. However, the direct differentiation of mES cells by treatment with CDCA without an EB step has not yet been studied. To determine the optimal concentration of CDCA for inducing differentiation of mES cells, we used 50, 100, and 200 uM CDCA to treat the mES cells for 72 h. In mES cells treated with 100 and 200 *μ*M CDCA, the pluripotent signals were reduced as the cells differentiated into ectodermal and mesodermal lineages. Concomitant results were shown for mRNA expression analysis by RT-PCR, demonstrating that CDCA can induce the differentiation of mES cells. However, endodermal differentiation was not observed. Since 200 *μ*M CDCA reduced the cell number as well as viability about 50% than 100 *μ*M CDCA, we chose to use a 100 *μ*M dose for the second passage. In the second round of treatment, differentiation of mES cells into ectodermal and endodermal lineages was more significant. The expression of pluripotent markers of ES cells such ALP, Oct4, and Nanog was almost absent at the protein level, and the mRNA levels of these markers were drastically reduced, while maintaining the cell viability. 

Our results indicate that CDCA treatment of undifferentiated mES cells causes differentiation by a pathway other than the one involved in adult organ regeneration and does not involve the FXR receptor. CDCA is a primary bile acid synthesized directly from cholesterol in the liver and secreted via the bile into the small intestine that plays a key role in the digestion and absorption of dietary fats [30]. CDCA-activated FXR regulates expression of genes whose products are critically important for bile acid and cholesterol homeostasis in cultivated hepatocytes [31, 32] and liver slices [33]. Moreover, several studies have shown that the FXR ligand, CDCA, can induce differentiation, inhibit proliferation, and induce the apoptosis of several primary cell types including human fibroblast [21] and keratinocytes [20]. Furthermore, CDCA can regulate the differentiation of mouse preadipocytes into mature adipocytes [23] and can mediate apoptosis in vascular smooth muscle cells and breast cancer cells [22, 24].

It is not reported whether there is an endogenous expression in embryonic stage but there are several reports that human fetus in early gestation (weeks 13–19) produces CDCA [34] and porcine fetus also does in even earlier stage during gestation (weeks 4) [35]. Recently, FXR-deficient mice showed that FXR may control adipocyte differentiation via PPAR-*γ* and Wnt/*β*-catenin pathways [36]. 

There have been several reports that CDCA can mediate effects in an FXR-independent regulatory manner. CDCA can mediate the activity of PKC [37, 38], which plays a key role in the regulation of cell growth, differentiation, and apoptosis [39]. Moreover, CDCA can directly activate the growth regulatory gene, *cyclooxygenase-2* [40], and transcription factors such as *c-Fos* [41] and *activator protein-1 (AP-1)* [42], which are involved in the regulation of cell growth and differentiation. Recent reports suggest that bile acid-mediated apoptosis is dependent on death receptor signaling [43] or mitochondria dysfunction [44]. Through activation of these diverse signaling pathways, CDCA can regulate several cellular activities. 

Although the exact mechanism of CDCA-induced differentiation of mES cells has to be elucidated, we have demonstrated that CDCA directly induces the differentiation of mES cells into ectodermal and mesodermal cells in a dose-dependent manner but does not promote endodermal differentiation. It would be also necessary to investigate the CDCA-induced differentiation for longer period to prove determination of their lineages to the ectodermal/mesodermal not endodermal cells in the near future.

Furthermore, CDCA-induced differentiation of mES cells seems to be mediated by an FXR-independent mechanism. In conclusion, these results provide useful information concerning the role of CDCA in the cellular activities of mES cells. However, determination of the exact mechanisms of CDCA-mediated antiproliferation and differentiation of mES cells requires further research.

## Supplementary Material

Supplementary Figure 1: Before direct differentiation of mES cells by CDCA, we maintained the cells in feeder-free condition. The suspended mES cells were once transferred onto a new 0.1% gelatin-coated dish for propagation in the presence of 1,000 U/ml of LIF and cultured for 4d. The cells on feeder-free condition expressed stem cell markers such as ALP, Oct4 and Nanog. Characterization of mES cells under feeder-free conditions. (b) in comparison with maintenance of mES cells on MEF feeder cells (a). mES cells showing alkaline phosphatase activity (c, d). Positive immunoreactivity with antibodies to Oct4 (e,f) and Nanog (g,h). mES cells, mouse embryonic stem cells; MEF, mouse embryonic fibroblast. Bar = 50 *μ*m.
Supplementary Figure 2: To investigate the direct effect of CDCA on mES cell, we examined morphology of CDCA-treated cells at 24, 48 and 72 h. Morphological changes in CDCA-treated mES cells under feeder-free conditions. CDCA 5050 *μ*M (g-i), 10050 *μ*M (j-l) and 20050 *μ*M (m-o) CDCA. mES cells, mouse embryonic stem cells; MEF, mouse embryonic fibroblast. Bar = 50 *μ*m.
Supplementary Figure 3: We analyzed FXR mRNA expression of CDCA-induced differentiated cells derived from ES cell if the phenomenon has been related with the nuclear receptor directly. The mRNA expression of FXR was not detected in controls and CDCA-treated cells. FXR mRNA expression in mES cells. LIF, leukemia inhibitory factor; DMSO, dimethyl sulfoxide; CDCA, chenodeoxycholic acid.

## Figures and Tables

**Table 1:** Primer sequences and cycling conditions used for RT-PCR.

Gene	Primer sequence	Annealing temperature	Product size
Oct4	Forward 5′-GAAGCCCTCCCTACAGCAGA-3′	60°C	297 bp
	Reverse 5′-CAGAGCAGTGACGGGAACAG-3′		
Nanog	Forward 5′-CCCCACAAGCCTTGGAATTA-3′	60°C	255 bp
	Reverse 5′-CTCAAATCCCAGCAACCACA-3′		
Nestin	Forward 5′-TAGAGGTGCAGCAGCTGCAG-3′	60°C	170 bp
	Reverse 5′-AGCGATCTGACTCTGTAGAC-3′		
NCAM	Forward 5′-AGATGGTCAGTTGCTGCCAA-3′	60°C	187 bp
	Reverse 5′-AGAAGACGGTGTGTCTGCTT-3′		
*α*-SMA	Forward 5′-ACTGGGACGACATGGAAAAG-3′	60°C	240 bp
	Reverse 5′-CATCTCCAGAGTCCAGCACA-3′		
Desmin	Forward 5′-TGACAACCTGATAGACGACC-3′	60°C	180 bp
	Reverse 5′-TTAAGGAACGCGATCTCCTC-3′		
*α*-FP	Forward 5′-TGCACGAAAATGAGTTTGGGA-3′	60°C	159 bp
	Reverse 5′-TTGCAGCCAACACATCGCTA-3′		
Albumin	Forward 5′-TGCTGCTGATTTTGTTGAGG-3′	60°C	500 bp
	Reverse 5′-GCTCACTCACTGGGGTCTTC-3′		
FXR	Forward 5′-TTGCGACAAGTGACCTCCAC-3′	58°C	653 bp
	Reverse 5′-TGATGGTTGAATGTCCGGAG-3′		
GAPDH	Forward 5′-GTCATCATACTTGGCAGGTT-3′	60°C	489 bp
	Reverse 5′-GTCGTGGAGTCTACTGGTGT-3′		

NCAM: neural cell adhesion molecule; *α*-SMA: alpha-smooth muscle actin; *α*-FP: alpha-fetoprotein; FXR: farnesoid X receptor; GAPDH: glyceraldehyde-3-phosphate dehydrogenase.
