# Supplementary figures and images for: Direct Effect of Chenodeoxycholic Acid on Differentiation of Mouse Embryonic Stem Cells Cultured under Feeder-Free Culture Conditions
Source: Biomed Res Int. 2012 Dec 29;2013:375076. doi: 10.1155/2013/375076 (PMC3591134; doi:10.1155/2013/375076)

# Supplement 1

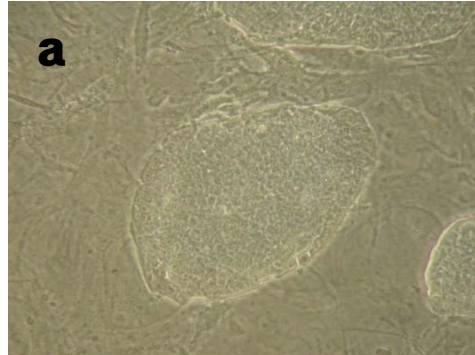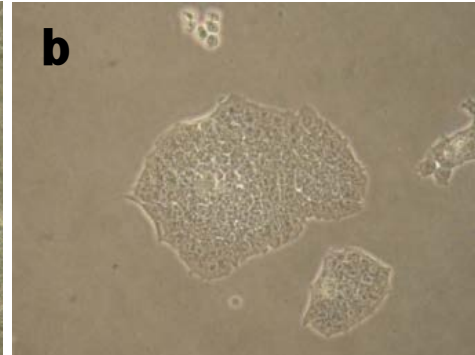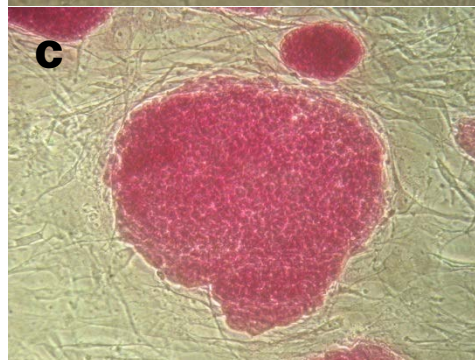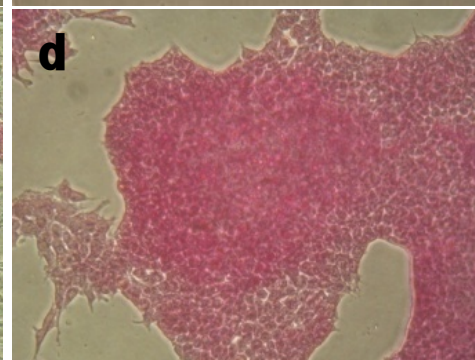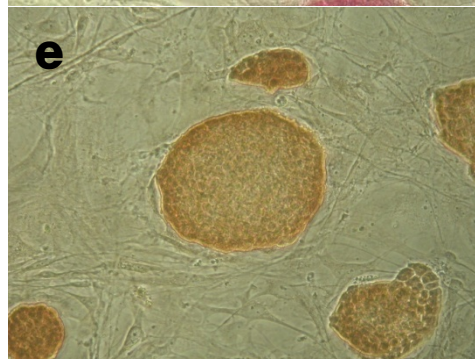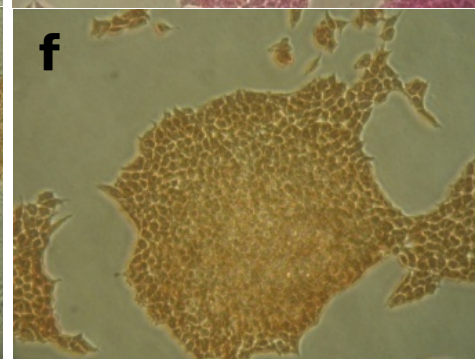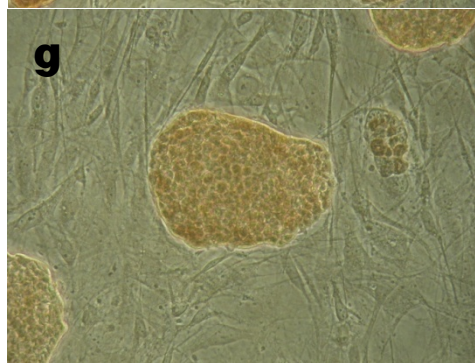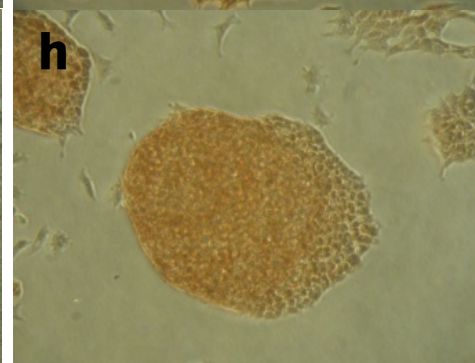

50  $\mu$ m

## Supplement 2

LIF

0.1% DMSO

50

100

200

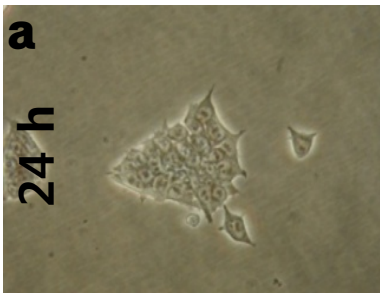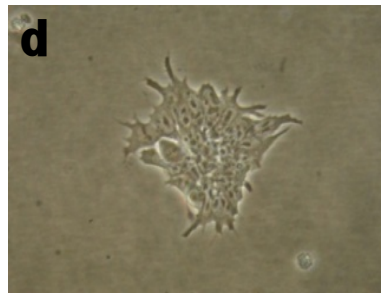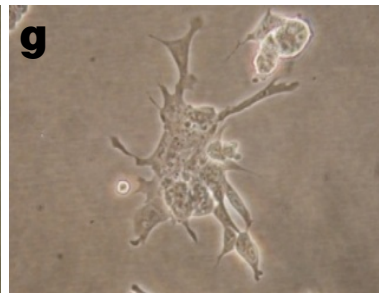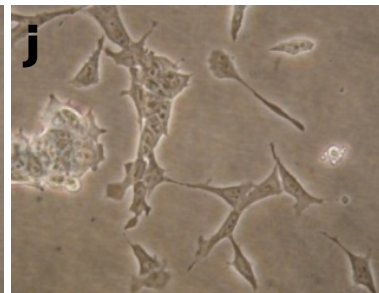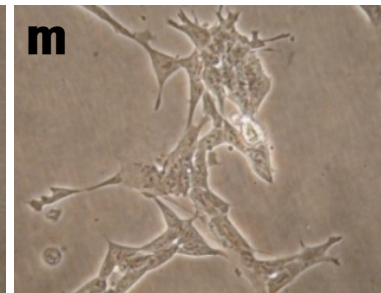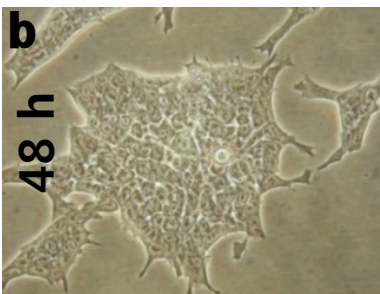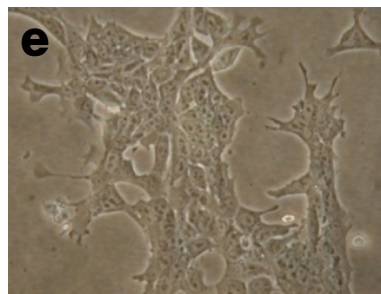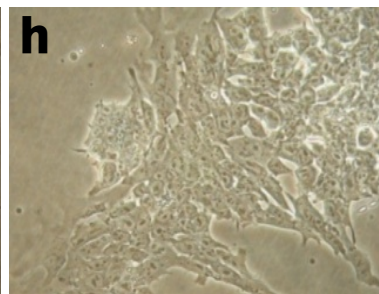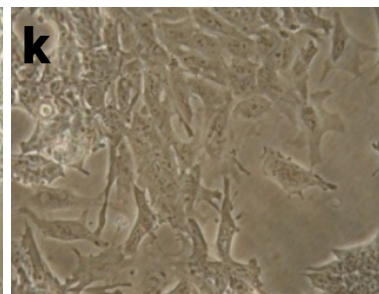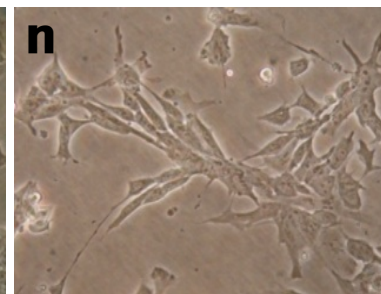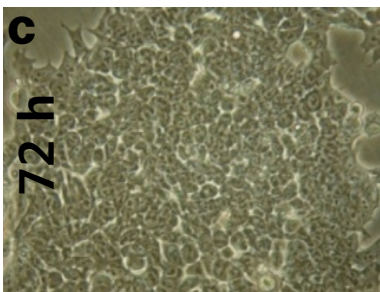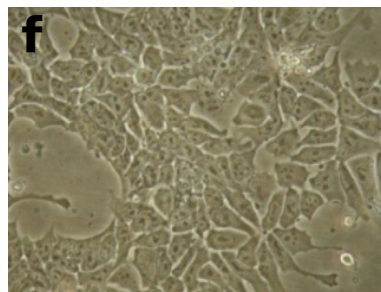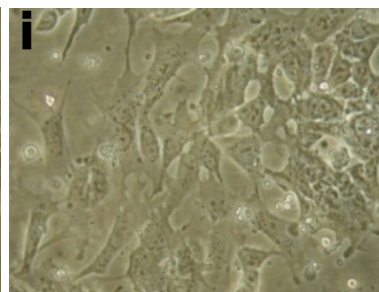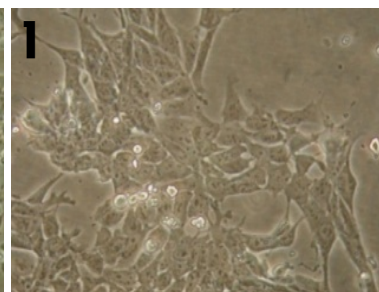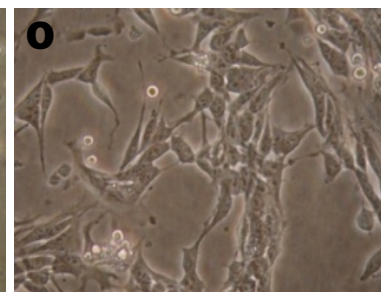

50  $\mu$ m

## Supplement 3

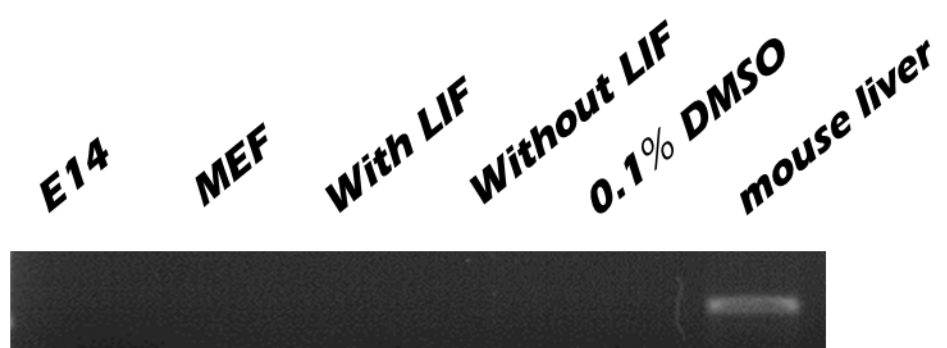

Supplement: Supplementary file 1 — Supplementary Figure 1: Before direct differentiation of mES cells by CDCA, we maintained the cells in feeder-free condition. The suspended mES cells were once transferred onto a new 0.1% gelatin-coated dish for propagation in the presence of 1,000 U/ml of LIF and cultured for 4d. The cells on feeder-free condition expressed stem cell markers such as ALP, Oct4 and Nanog. Characterization of mES cells under feeder-free conditions. (b) in comparison with maintenance of mES cells on MEF feeder cells (a). mES cells showing alkaline phosphatase activity (c, d). Positive immunoreactivity with antibodies to Oct4 (e,f) and Nanog (g,h). mES cells, mouse embryonic stem cells; MEF, mouse embryonic fibroblast. Bar = 50 μm. Supplementary Figure 2: To investigate the direct effect of CDCA on mES cell, we examined morphology of CDCA-treated cells at 24, 48 and 72 h. Morphological changes in CDCA-treated mES cells under feeder-free conditions. CDCA 5050 μM (g-i), 10050 μM (j-l) and 20050 μM (m-o) CDCA. mES cells, mouse embryonic stem cells; MEF, mouse embryonic fibroblast. Bar = 50 μm. Supplementary Figure 3: We analyzed FXR mRNA expression of CDCA-induced differentiated cells derived from ES cell if the phenomenon has been related with the nuclear receptor directly. The mRNA expression of FXR was not detected in controls and CDCA-treated cells. FXR mRNA expression in mES cells. LIF, leukemia inhibitory factor; DMSO, dimethyl sulfoxide; CDCA, chenodeoxycholic acid. [file 375076.f1.pdf]
